# Trends in Utilization and Costs Following a Hepatitis C Elimination Initiative

**DOI:** 10.1001/jamanetworkopen.2025.58714

**Published:** 2026-02-10

**Authors:** Ashley Tabah, Anirban Basu, Paula Cox-North, Judy Zerzan-Thul, Leta Evaskus, Donna Sullivan, JoEllen Colson, Emalie Huriaux, Jon Stockton, Omeid Heidari, Lisa Wiggins, Stella Chang, Pamela Kohler

**Affiliations:** 1 The Comparative Health Outcomes, Policy, and Economics (CHOICE) Institute, University of Washington, Seattle; 2 Department of Biobehavioral Nursing and Health Informatics, University of Washington, Seattle; 3 Washington State Health Care Authority, Olympia; 4 Washington State Department of Health, Tumwater; 5 Division of Allergy and Infectious Diseases, University of Washington, Seattle; 6 Department of Child, Family, and Population Health Nursing, University of Washington, Seattle; 7 Department of Global Health, University of Washington, Seattle

## Abstract

**Importance:**

Expanded screening and treatment options as part of national guidelines and statewide policy initiatives have the potential to eliminate hepatitis C virus (HCV).

**Objective:**

To describe trends in HCV screening, prevalence, treatment, and costs before and after Washington State’s HCV elimination initiative.

**Design, Setting, and Participants:**

This case series is an observational analysis of retrospective claims data aggregated at the individual-month level. The dataset included individuals in the Washington State All Payers Claims Database between January 2017 and September 2022. Analyses were conducted from August to November 2025.

**Exposures:**

In July 2019, as part of a directive from the governor, the Washington State Department of Health released a comprehensive HCV elimination strategy. The plan included discounted and subscription-based payment models and removal of restrictive prior authorization and specialist consultation requirements.

**Main Outcomes and Measures:**

*International Statistical Classification of Diseases and Related Health Problems, Tenth Revision (ICD-10) *and *Current Procedural Terminology* codes were used to identify HCV testing or screening, *ICD-10* diagnosis codes were used for HCV infections, and the National Drug Codes were used for HCV direct-acting antiviral medications. An individual was considered to have newly diagnosed HCV at the earliest month in which they had an HCV test claim followed by 2 HCV diagnosis codes within the subsequent 12 months and no HCV diagnosis during the 6 months before the test month. Total health care costs per month were computed, excluding cost of HCV direct-acting antiviral medications. Interrupted time-series approaches computed trends over time using the second quarter (Q2) of 2019 as a reference time point.

**Results:**

More than 21 million unique individuals (mean [SD] age, 44 [26] years; 11 085 080 female [53%]) were included in the data extraction. The number of HCV tests per month was stable during the preinitiative and immediate postinitiative period. Beginning in 2020 Q3, screening increased significantly from the reference quarter by 10.5 additional tests per month per 1000 Washington enrollees (95% CI, 8.8 to 12.2 tests per month per 1000 enrollees; *P* < .001). Consistent with increased screening, the prevalence of chronic HCV cases increased through 2021, but declined by 0.4 cases per 1000 enrollees comparing 2019 Q2 to 2022 Q3 (95% CI, −0.6 to −0.2 cases per 1000 enrollees; *P* < .001). The proportion of newly diagnosed cases receiving any HCV treatment increased from 16.8% (634 of 3776 cases) in 2017 to 24.9% (472 of 1894 cases) in early 2021 (difference, 8.1 percentage points; 95% CI, 5.8 to 10.4 percentage points; *P* < .001), but slopes before and after 2019 Q2 were not significantly different. Costs per ever prevalent case decreased significantly by 2021 Q1 (−$520 per month compared with 2019 Q2; 95% CI, −$922 to −$124 per month; *P* = .01).

**Conclusions:**

In this longitudinal case series analysis of claims data, Washington State’s elimination initiative, combined with revised national screening guidelines and improved treatment options, contributed to expansion of HCV screening and changes over time in the proportion of people identified and treated for HCV, as well as decreased overall care costs.

## Key Points

**Question:**

What are the trends in hepatitis C virus (HCV) screening, diagnosis, treatment, and cost before and following implementation of a statewide HCV elimination initiative?

**Findings:**

This case series analysis of state claims data for more than 21 million unique individuals found that the number of HCV tests per month in Washington increased substantially 1 year after implementation, with prevalence increasing from 2017 to 2021 before declining significantly during 2022. Total costs for care increased from 2017 through 2019, consistent with higher prevalence during this period, but declined after mid-2020, even when prevalence was increasing.

**Meaning:**

These findings suggest that expanded screening and access to treatment may decrease costs of HCV care over time.

## Teaser

This case series examines trends in hepatitis C virus screening, prevalence, treatment, and costs before and after Washington State’s hepatitis C virus elimination initiative.

## Introduction

Hepatitis C virus (HCV) is the most common bloodborne infection in the US, with an estimated prevalence of 39.8 chronic hepatitis C cases per 100 000 people nationally and 51.5 chronic hepatitis C infections per 100 000 persons living in Washington State in 2021.^1^ Until 1990, HCV was an incurable illness and the leading cause of liver disease, with substantial mortality and financial burdens.^2^ As treatment for HCV has become available, states are challenged to identify low-cost strategies to rapidly identify and treat on a population-wide scale, including marginalized populations and those with limited resources or access to care.

In July 2019, as part of a directive from Governor Jay Inslee to eliminate the public health threat of HCV by the year 2030, the Washington State Department of Health released a comprehensive strategy, *Hep C Free Washington: Plan to Eliminate Hepatitis C in Washington State by 2030*. The plan called for combined public health approaches with a new medication purchasing approach, and included goals in program coordination, surveillance and reporting, community-based responses and interventions, and clinical strategies.^3^

To implement this plan, the Washington State Health Care Authority (HCA) contracted with a manufacturer of HCV direct-acting antiviral (DAA) medication.^3^ HCA signed 2 contracts with the manufacturer of Mavyret (glecaprevir-pibrentasvir). For non-Medicaid state covered lives, a discounted price was negotiated for the HCV DAA medication. For Washington Apple Health (Medicaid) lives, HCA negotiated a novel modified subscription-based payment model that includes a discounted price for the DAA medication and a treatment goal that, once met, dramatically reduces the price. Although the terms of these contracts are not public, the goal is to treat more lives with the same budget. Additionally, HCA updated the Apple Health HCV treatment policy to remove previous restrictions, including removing both the necessity for prior authorization for the contracted HCV DAA medication and the requirement for specialist consultation to treat Apple Health clients.^4^ In March 2020, national guidelines were additionally expanded to recommend onetime screening for asymptomatic individuals regardless of risk factors. This article aims to describe time trends and outcomes, including HCV screening, prevalence, treatment initiation rates, and cost before and after implementation of the state’s elimination initiative.

## Methods

### Design

This is a retrospective, longitudinal case series analysis of Washington State claims data. This study received approval and a waiver of consent from the Washington State and the University of Washington institutional review boards. Strengthening the Reporting of Observational Studies in Epidemiology (STROBE) reporting guidelines for case series studies are followed in this report.

### Data Source

We used the Washington State All-Payer Claims Database (APCD), which includes approximately 6 to 8 million individuals receiving care each year in Washington State between January 2017 and September 2022.^5^ The Washington State APCD includes medical, pharmacy, and dental claims, plus eligibility and practitioner files collected from private and public payers, representing approximately 70% of the Washington State population plus an additional 2 to 3 million out-of-state members accessing care in Washington.^6^ During the reported time period, products held by unique members comprised approximately 40% commercial preferred provider organization or health maintenance organization plans, 30% Medicare, and 25% Medicaid.^7^ Medicaid Fee-for-Service was not included in the extraction. Data were aggregated at the individual-month level to conduct a descriptive analysis of HCV testing, new diagnoses, prevalence, medication use, and total health care cost every month. Because the costs of HCV DAA medications were carved out of reimbursements for Apple Health and Uniform Medical Plan, these costs were not included in the total health care cost estimates.

### Outcomes

We used *International Statistical Classification of Diseases and Related Health Problems, Tenth Revision (ICD-10)* and *Current Procedural Terminology* codes to identify HCV testing or screening, *ICD-10* diagnosis codes for hepatitis C infections, and the National Drug Codes for the HCV DAA medications (eTables 1-3 in Supplement 1). An individual was considered to have a new HCV diagnosis at the earliest month in which they had an HCV test claim (*ICD-10* and *Current Procedural Terminology* codes; eTable 2 in Supplement 1) followed by 2 HCV diagnosis codes within the subsequent 12 months, including month of test, and no HCV diagnosis during the 6 months before the test month.^8^ Individuals meeting these criteria were deemed to have prevalent chronic HCV following the month of diagnosis. Individuals who were not defined as new cases during our data window were considered to have prevalent HCV if they had at least 2 HCV diagnosis codes within 12 months, starting from the first instance of the HCV diagnosis code. According to our definitions, we identified a total number of 7420 newly diagnosed hepatitis C cases in 2018, 5646 new cases in 2019, and 5196 new cases in 2020; the Washington State Department of Health reported about 7500 newly diagnosed chronic cases in 2018, 6500 cases in 2019, and 5000 chronic cases in 2020.^9^

Antivirals for treatment included glecaprevir-pibrentasvir (DAA medication contracted by the state for Medicaid and non-Medicaid state-covered lives), sofosbuvir-velpatasivir, sofosbuvir-velpatasvir-voxilaprevir, ledipasvir-sofosbuvir, and elbasvir-grazoprevir. Upon completion of a medication’s guideline-recommended duration of therapy, individuals with prevalent HCV were deemed cured and exited the prevalent group. Once cured, these individuals were re-evaluated for incident HCV per the aforementioned definition. Incident cases were defined as treated if any treatment was identified within 6 months of the diagnosis month. Total health care costs include costs related to all health care services except for the aforementioned medications.

### Statistical Analysis

Analyses were conducted from August to November 2025 using R statistical software version 4.2.2 (R Project for Statistical Computing). We considered each month’s data a cross-sectional panel of individuals in Washington State. We conducted a descriptive trend analysis of the outcomes with Washington State’s elimination initiative implementation date of July 1, 2019. Results were reported between July 2017 and September 2022, with the exception of new case data, which were calculated up to December 2021. Cost data were adjusted for inflation. Trends were assessed using interrupted time-series approaches. In most cases, we evaluated interrupted time-series in discrete time by testing the changes in the average monthly outcome in each quarter (Q) from 2019 Q2 (policy start) as the base quarter. For treatment uptake trends that were linear, we were able to fit a disjointed linear regression to compute changes in slope for treatment uptake over time among newly diagnosed cases. We adjusted for the corresponding monthly denominator in every regression. Huber-White robust SEs were used. Both approaches used a 2-sided *t* test, and statistical significance was assessed using 95% CIs, with significance defined as a 95% CI that did not include the null value of zero and *P* < .05.

## Results

### APCD Cohort

The APCD extract included records from more than 21 million unique individuals. The mean (SD) age was 44 (26) years; 11 085 080 individuals (53%) were female, 9 712 405 (46%) were male, and 293 310 (1%) were unknown gender. For the purpose of this analysis, we restricted to medical claims identified from 5 608 952 individuals in 2017, 5 571 805 individuals in 2018, 6 257 739 individuals in 2019, 7 120 974 individuals in 2020, and 7 560 496 individuals in 2021.

### HCV Screening

**Figure 1.**
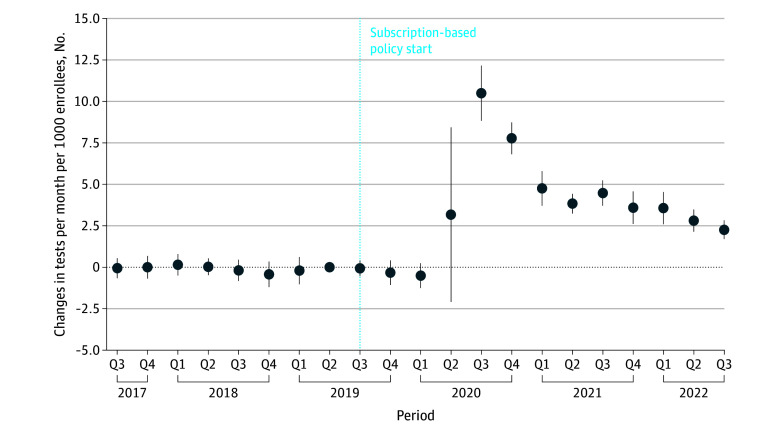
Change in Counts of Hepatitis C Virus Tests Administered in Washington State The reference quarter (Q) is 2019 Q2. Circles denote means, and error bars denote 95% CIs. Data were obtained from the Washington State All Payers Claims Database, 2025.

The average monthly number of HCV tests in Washington was stable during the period prior to the implementation of the elimination initiative. There was a median (IQR) of 28 375 (26 457-30 490) tests per month in the latter half of 2017 and 28 647 (26 840-30 316) tests per month in 2018 (eFigure 1 in Supplement 1). These counts remained stable for a year after implementation from 30 062 in July 2019 to 24 165 in April 2020. Beginning in 2020 Q3, test counts increased significantly from the reference quarter (2019 Q2) to a peak of 99 161 in July 2020 (an increase by 10.5 additional tests per month per 1000 enrollees from 2019 Q2 to 2020 Q3; 95% CI, 8.8-12.2 tests per month per 1000 enrollees; *P* < .001) (Figure 1). This constituted a 200% increase from the baseline use of approximately 5 tests per month per 1000 enrollees in 2019 Q2. Counts then remained high during 2020 (median [IQR], 74 181 [29 146-81 688] tests per month) before leveling off at a median (IQR) of 55 844 (53 655-60 776) tests per month in 2021.

### Prevalence

Among all beneficiaries in the APCD each month, the prevalence count of chronic HCV cases over 6-month periods increased from 22 596 of 5 497 616 cases (0.4%) in January to June 2017 to 40 906 of 6 130 414 cases (0.7%) in 2021 (Table). The mean (SD) age of these cases was 52 (14) years, with 10 041 (44%) being female. Demographics remained stable over the years studied. Prevalent cases continued to increase linearly and significantly before and following the reference quarter through 2021 and began to decline by late 2022 (0.4 cases per 1000 enrollees lower in 2022 Q3 than in 2019 Q2; 95% CI, −0.6 to −0.2 cases per 1000 enrollees; *P* < .001) (eFigure 2 in Supplement 1).

**Table.** Prevalence, New Cases, and Treatment of HCV in the Washington State All Payers Claims Database, 6-Month Intervals

Time	Population size represented, No. of individuals	HCV tests, No.	Any HCV treatment among newly diagnosed cases, No./total No. (%)^a^	HCV prevalence			
				Case count, No. (%)	Age, mean (SD), y	Female sex, No. (%)	Male sex, No. (%)
July-December 2017	5 497 616	151 103	634/3776 (16.8)	22 596 (0.4)	52.6 (13.5)	10 041 (44.4)	12 555 (55.6)
January-June 2018	5 570 982	155 292	571/3574 (16.0)	27 980 (0.5)	52.4 (13.8)	12 449 (44.5)	15 531 (55.5)
July-December 2018	5 563 051	143 850	396/2494 (15.9)	31 282 (0.6)	52.3 (14.0)	13 919 (44.4)	17 363 (55.5)
January-June 2019	5 662 250	151 693	462/2404 (19.2)	33 944 (0.6)	52.3 (14.2)	15 067 (44.4)	18 877 (55.6)
July-December 2019	5 691 306	149 327	505/2210 (22.6)	35 863 (0.6)	52.4 (14.3)	15 958 (44.5)	19 905 (55.5)
January-June 2020	5 901 222	204 648	374/2011 (18.6)	37 683 (0.6)	52.6 (14.5)	16 702 (44.3)	20 981 (55.7)
July-December 2020	5 870 558	419 163	401/2094 (19.2)	39 306 (0.7)	52.8 (14.6)	17 406 (44.3)	21 900 (55.7)
January-June 2021	6 078 818	291 178	472/1894 (24.9)	40 825 (0.7)	53.0 (14.6)	18 082 (44.3)	22 743 (55.7)
July-December 2021	6 130 414	284 559	237/1130 (21.0)	40 906 (0.7)	53.2 (14.7)	18 151 (44.4)	22 755 (55.6)

Abbreviation: HCV, hepatitis C virus.

^a^ Refers to any treatment within 6 months.

### New Diagnoses

**Figure 2.**
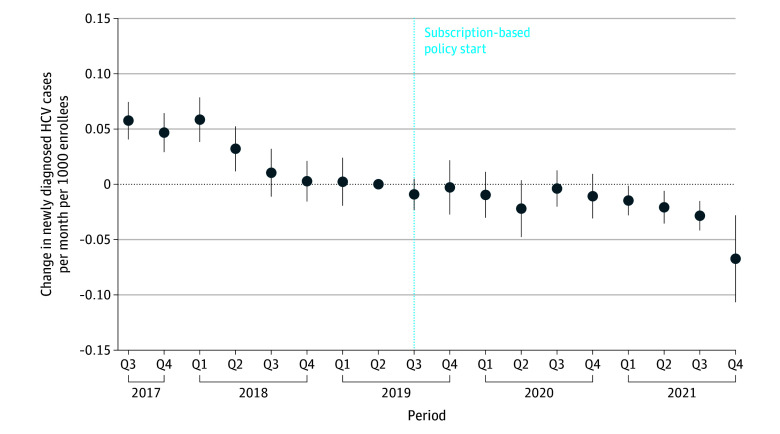
Change in Counts of New Hepatitis C Virus (HCV) Diagnoses The reference quarter (Q) is 2019 Q2. Circles denote means, and error bars denote 95% CIs. Data were obtained from the Washington State All Payers Claims Database, 2025.

The median (IQR) number of newly diagnosed cases was 759 (709 to 786) per month during the latter half of 2017. Beginning in 2018, counts began declining by 14 to 16 cases per month over the next year, with a median (IQR) of 582 (521 to 716) cases per month (Figure 2). Since the implementation of the state’s elimination initiative in July 2019, new monthly cases continued to decline, but were not significantly lower, with a median (IQR) of 448 (379 to 501) cases per month in 2020. In the first 6 months of 2021, cases began to decline further, with a median (IQR) of 419 (375 to 430) cases per month, a significant decline from the reference quarter (decrease of 0.02 new cases per month per 1000 enrollees from 2019 Q2 to 2021 Q1; 95% CI, −0.035 to −0.005 new cases per month per 1000 enrollees; *P* = .03).

### Treatment

**Figure 3.**
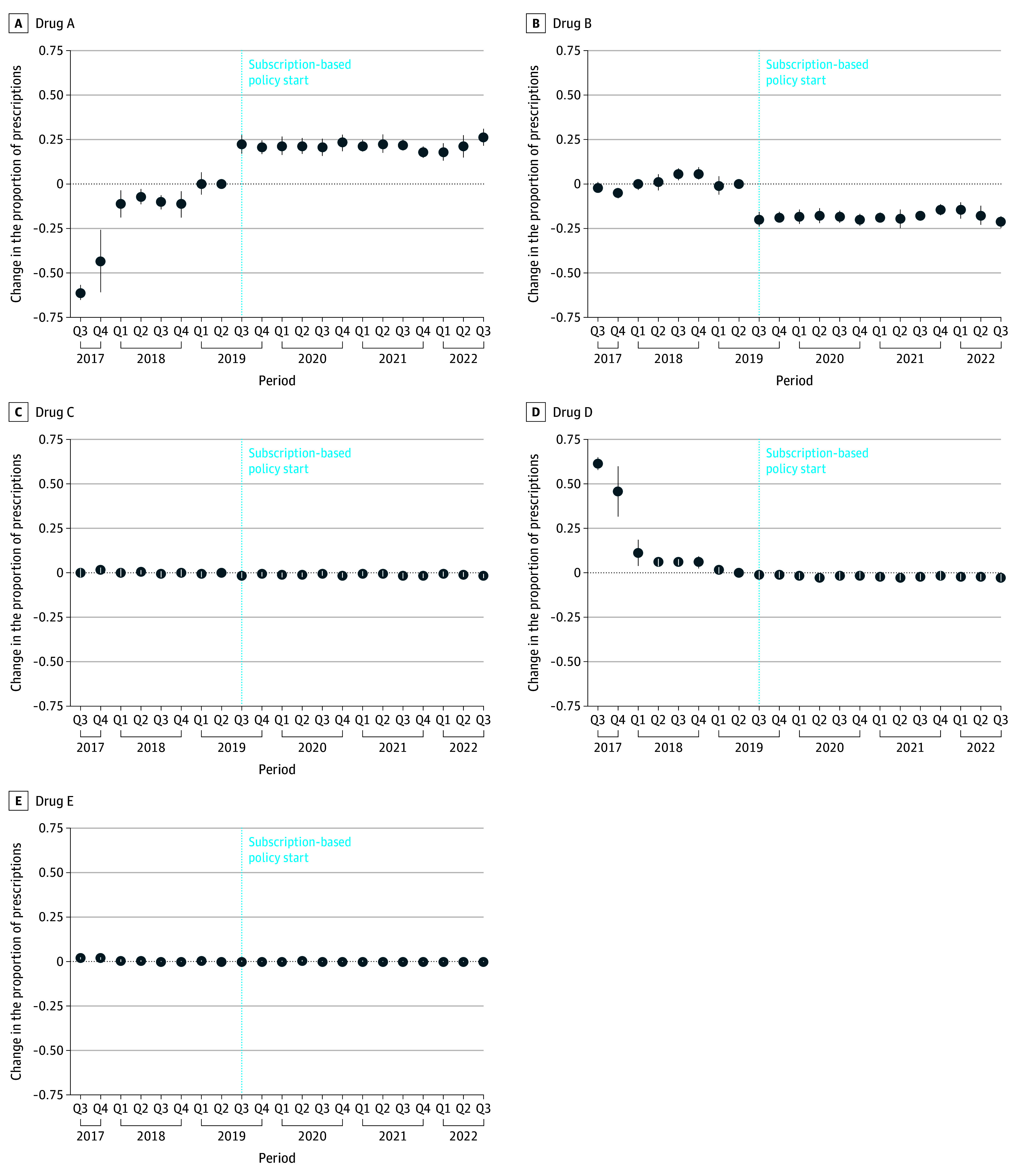
Change in Proportion of Specific Medications for Hepatitis C Virus Treatment in Washington State The reference quarter (Q) is 2019 Q2. Circles denote means, and error bars denote 95% CIs. Data were obtained from the Washington State All Payers Claims Database, 2025. Drug A is glecaprevir-pibrentasvir, drug B is sofosbuvir-velpatasivir, drug C is sofosbuvir-velpatasvir-voxilaprevir, drug D isledipasvir-sofosbuvir, and drug E is elbasvir-grazoprevir.

eFigure 3 in Supplement 1 shows the change in number of curative treatments administered per month compared with the reference month. These numbers declined every month before the elimination initiative implementation and continued declining after implementation, except for a small increase (up to approximately 1000 prescriptions in July 2019) immediately after implementation. Most of the increase in prescriptions was associated with the contracted DAA medication (Figure 3). There was a sharp increase in use in the first month of program implementation in July 2019 and remained slightly elevated in the subsequent months after the start of the contract, compared with precontract levels. Use of all other DAA medications showed a steady decline over this period.

Approximately 1% to 2% of prevalent cases with HCV infections in the state per month received any prescription for a specific HCV DAA medication. Accounting for the fact that some individuals received more than 1 prescription per month, the average proportion of the prevalent population receiving any HCV DAA medications per month was approximately 0.5% (150 of 31 261 patients) in June 2019. This proportion increased to approximately 1.4% (462 of 31 895 patients) right after the start of the contract in August 2019 but came down to the precontract levels by June 2020. Total corresponding annual treatment numbers among incident cases are shown in the Table. The proportion of incident cases receiving any HCV treatment within 6 months increased from 16.8% (634 of 3776 cases) in 2017 to 24.9% (472 of 1894 cases) in early 2021 (difference, 8.1 percentage points; 95% CI, 5.8-10.4 percentage points; *P* < .001). Although steadily increasing over time, the slope after policy implementation was not significantly different than before 2019 Q3. By 2021, 85% (1404 of 1645 patients) of any patients (incident or prevalent) taking any HCV DAA medication received glecaprevir-pibrentasvir.

### Health Care Costs

**Figure 4.**
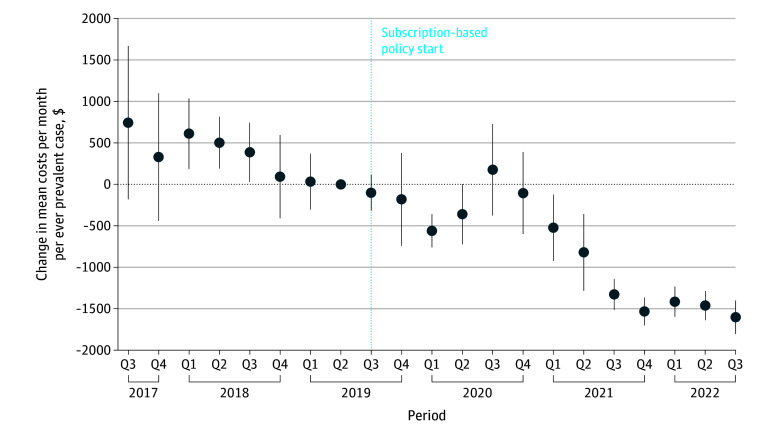
Change in Per-Patient Mean Total Health Care Costs (Without Hepatitis C Virus Direct-Acting Antiviral Medication Costs) The reference quarter (Q) is 2019 Q2. Circles denote means, and error bars denote 95% CIs. Data were obtained from the Washington State All Payers Claims Database, 2025.

Total monthly costs of care increased from $45.6 million in 2017 to $70.8 million in 2019, presumably owing to the increase in the prevalence population in the state during this period (eFigure 4 in Supplement 1). However, total costs appear to decrease from June 2020, even when prevalence kept increasing. Total monthly costs of care were $58.6 million in 2021. The mean per-patient monthly costs declined from $2824 in 2017 to $2179 in 2019 (a 22.8% decline). By 2021, mean per-patient monthly costs were significantly lower than the reference quarter (45.4% decline from 2019; decrease by $520 per month per ever-prevalent case from 2019 Q2 to 2021 Q1; 95% CI, −$922 to −$124 per month; *P* = .01) (Figure 4).

## Discussion

This case series analysis used a routine claims dataset from the State of Washington to assess longitudinal trends in HCV prevalence, testing, treatment, and cost during months before and after the implementation of Washington State’s elimination initiative. We identified a significant increase in HCV screening in the months following initiative implementation. However, the spike in testing started from June 2020 and could be more aligned with the release of revised national guidelines that all adults receive 1 screening test regardless of risk factors.^10^ This nearly 500% increase in screening suggests rapid adoption by clinical practitioners. Prior research additionally suggests that targeted efforts in community and public health settings may further contribute to expanded coverage of HCV testing.^11^ Screening rates were not sustained over time, plummeting over the following 12 months and settling around June 2021 higher than the preinitiative rates, likely as lower-risk individuals obtained their onetime screening.

Applying established clinical definitions to the APCD database, we found estimated 5370 new cases per year, which closely resembled the HCV surveillance numbers reported by the Washington State Department of Health, with the exception of 2019, which identified approximately 850 fewer cases.^10^ New cases declined starting from before the initiative but showed signs of slowing down immediately after. In the context of a large expansion of screening that includes lower risk populations, such slowing down of the decline of new diagnoses could suggest improved reach to asymptomatic individuals without a documented history of risk behavior.

Use of contracted HCV DAA medications spiked immediately after the contract started and settled at a level slightly higher than precontract levels. During the same time, the use of noncontracted DAA medications continued to decline. Access to treatment improved over time among incident cases, although these numbers are lower and perhaps more conservative than reported state treatment rates, likely because of the restriction to treatment within 6 months of diagnosis. Average health care costs (excluding HCV medication costs) per ever-prevalent patient with HCV have declined since about 1 year after the start of the contract compared with precontract trends. This decline may be due to increased screening and detection of infections in otherwise healthy individuals, with improved curative outcomes and fewer comorbidities over time.^12^

### Limitations

The APCD database complements the state’s passive public health surveillance system but has some limitations for tracking true incidence and treatment rates. It may not have captured all completed tests, as some were delivered via nonclinical community-based testing programs and to those without health insurance. Moreover, the data reported here do not include diagnosis and tests conducted in correctional and other state facilities. Because the individuals in the APCD represent insured lives, those not represented in the APCD may be disproportionately less likely to access care and be at higher risk for loss to follow-up after diagnosis. Furthermore, our analysis does not make any claim on effect attributable to Washington State’s elimination initiative, as other longitudinal factors may have contributed. The COVID-19 pandemic had broad impacts on uptake of health services during the observational time period, although reassuringly, it would have biased outcomes toward the null as the pandemic occurred after implementation of the elimination plan.

## Conclusions

The findings from this longitudinal case series analysis of claims data suggest that Washington State’s elimination initiative, combined with revised national screening guidelines and improved treatment options, has contributed to significant expansion of HCV screening and changes over time in the proportion of people identified and treated for HCV. Decreasing costs per person suggest that screening and curative treatment goals are imperative.

## Notes

Supplement 1. eTable 1. *ICD-10* and *CPT* codes used to identify HCV screening eTable 2. *ICD-10* diagnosis code for HCV infections eTable 3. NDC codes for antiviral medications for HCV eFigure 1. Monthly counts of HCV tests administered in Washington state eFigure 2. Change in prevalent cases per 1000 enrollees compared to reference quarter (2019Q2) eFigure 3. Monthly counts of curative treatment administered in Washington state eFigure 4. Per-patient average total health care costs (without HCV direct-acting antiviral medication costs)

Supplement 2. Data Sharing Statement
