# Supplementary material for: Trends in Utilization and Costs Following a Hepatitis C Elimination Initiative
Source: JAMA Netw Open. 2026 Feb 10;9(2):e2558714. doi: 10.1001/jamanetworkopen.2025.58714 (PMC12892153; doi:10.1001/jamanetworkopen.2025.58714)
Supplement: Supplement 1. — eTable 1. ICD-10 and CPT codes used to identify HCV screening eTable 2. ICD-10 diagnosis code for HCV infections eTable 3. NDC codes for antiviral medications for HCV eFigure 1. Monthly counts of HCV tests administered in Washington state eFigure 2. Change in prevalent cases per 1000 enrollees compared to reference quarter (2019Q2) eFigure 3. Monthly counts of curative treatment administered in Washington state eFigure 4. Per-patient average total health care costs (without HCV direct-acting antiviral medication costs) [file jamanetwopen-e2558714-s001.pdf]

Supplemental Online Content

Tabah A, Basu A, Cox-North P, et al. Trends in utilization and costs following a hepatitis C elimination initiative. *JAMA Netw Open*. 2026;9(2):e2558714. doi:10.1001/jamanetworkopen.2025.58714

eTable 1. *ICD-10* and *CPT* codes used to identify HCV screening
eTable 2. *ICD-10* diagnosis code for HCV infections
eTable 3. NDC codes for antiviral medications for HCV
eFigure 1. Monthly counts of HCV tests administered in Washington state
eFigure 2. Change in prevalent cases per 1000 enrollees compared to reference quarter (2019Q2)
eFigure 3. Monthly counts of curative treatment administered in Washington state
eFigure 4. Per-patient average total health care costs (without HCV direct-acting antiviral medication costs)

This supplemental material has been provided by the authors to give readers additional information about their work.

© 2026 Tabah A et al. *JAMA Network Open*.

eTable 1: *ICD-10* and *CPT* codes used to identify HCV screening

ICD-10 Codes	Description
Z20.5	Suspected exposure
Z11.59	Need for hepatitis C screening test
	Encounter for hepatitis C screening test for low-risk patient
Z11.59, Z91.89	Encounter for hepatitis C virus screening test for high-risk patient
Z13.818	Screening for hepatitis
R76.8	Positive hepatitis C antibody test / Hepatitis C antibody positive in blood
Z78.9	Hepatitis C antibody test negative
Z86.19	History of hepatitis C / History of positive hepatitis C / Hx of hepatitis C / received kidney from donor with hepatitis C/ History of hepatitis C virus infection / Hepatitis C virus infection cured after antiviral drug therapy / Hepatitis C virus infection resolved after antiviral drug therapy
**CPT Codes**	**Description**
86803	Hepatitis C antibody
86804	Hepatitis C Virus Antibody Confirmation, Serum
3266F	Hepatitis C, genotype test
87520	Hepatitis C RNA; direct probe technique
87521	Hepatitis C RNA; amplified probe technique
87522	Hepatitis C RNA; quantification
87902	Hepatitis C virus genotype analysis
G0472 (rapid test)	HCV antibody positive

© 2026 Tabah A et al. *JAMA Network Open*.

eTable 2: ICD-10 diagnosis code for HCV infections

ICD-10 Codes	Condition
B17.10	Acute hepatitis C virus infection without hepatic coma
B17.11	Acute viral hepatitis C with hepatic coma
B18.2	Chronic viral hepatitis C
B19.20	Unspecified viral hepatitis C without hepatic coma
B19.21	Unspecified viral hepatitis C with hepatic coma
K73.9 or K73.2 or K73.0 or K75.4 or T86.898 or Z94.83	Hepatitis, chronic persistent<br>Hepatitis, chronic, aggressive<br>Received pancreas from donor with hepatitis C

eTable 3: NDC codes for antiviral medications for HCV

Drug Type	NDC Code	Antiviral	Notes
**1**	0074-2625<br>00074-2600	glecaprevir/pibrentasvir (Mavyret™)	WA state contract is with this medication. Recommended duration of use: 8-12 weeks. Used for 12 weeks in those with cirrhosis
**2**	61958-2201<br>61958-2203<br>61958-2204<br>61958-2205	sofosbuvir/velpatasivir (Epclusa™)	Recommended duration of use: 12 or 24 weeks. 24 weeks in those with severe cirrhosis
**3**	61958-2401	sofosbuvir/velpatasvir/voxilaprevir (VOSEVI™)	Recommended duration of use: 12 weeks
**4**	61958-1801<br>61958-1803<br>61958-1804<br>61958-1805	ledipasvir/sofosbuvir (Harvoni™)	Recommended duration of use: 8 or 12 weeks.
**5**	0006-3074	elbasvir-grazoprevir (Zepatier™)	Not used anymore
**6**	Many	ribavirin(Copegus, Rebetol, Ribasphere)	Booster is never given alone but in conjunction with another med.

© 2026 Tabah A et al. *JAMA Network Open*.

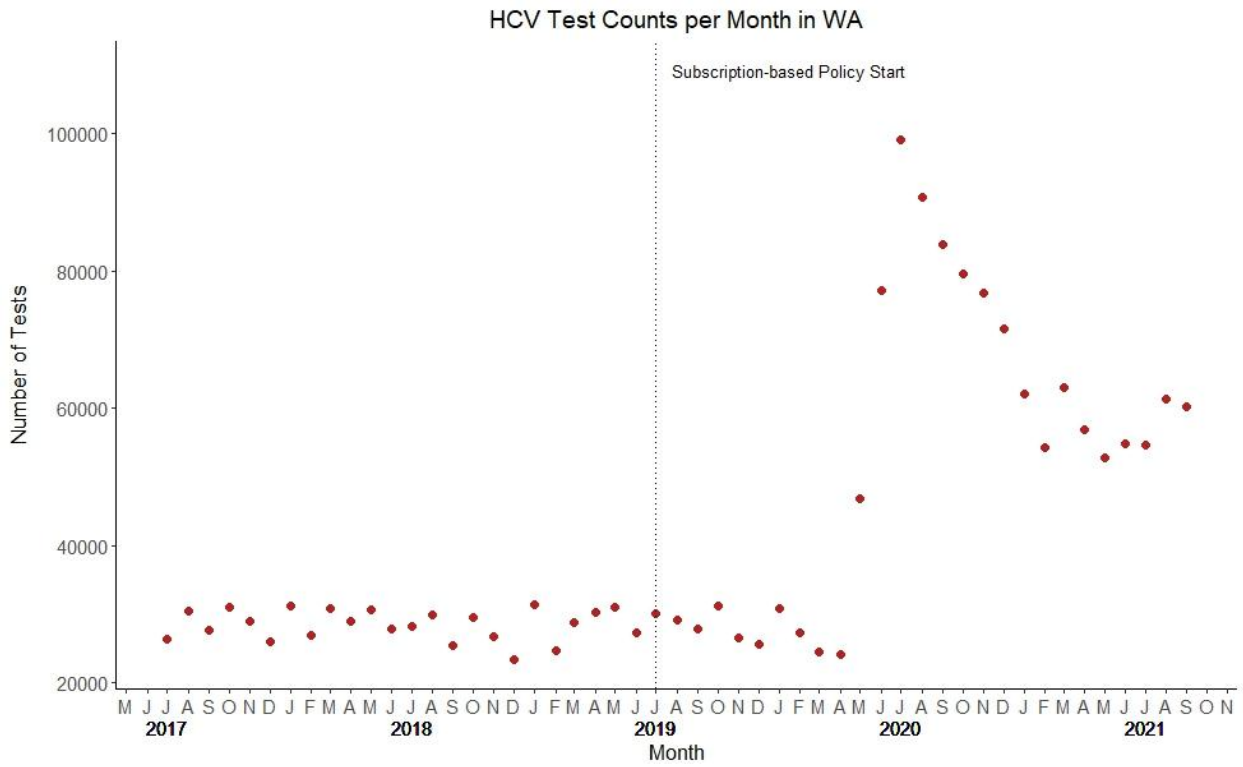

eFigure 1. Monthly counts of HCV tests administered in Washington state
Source - Authors' analysis of data from the Washington State All Payers Claims Database, 2025

© 2026 Tabah A et al. *JAMA Network Open*.

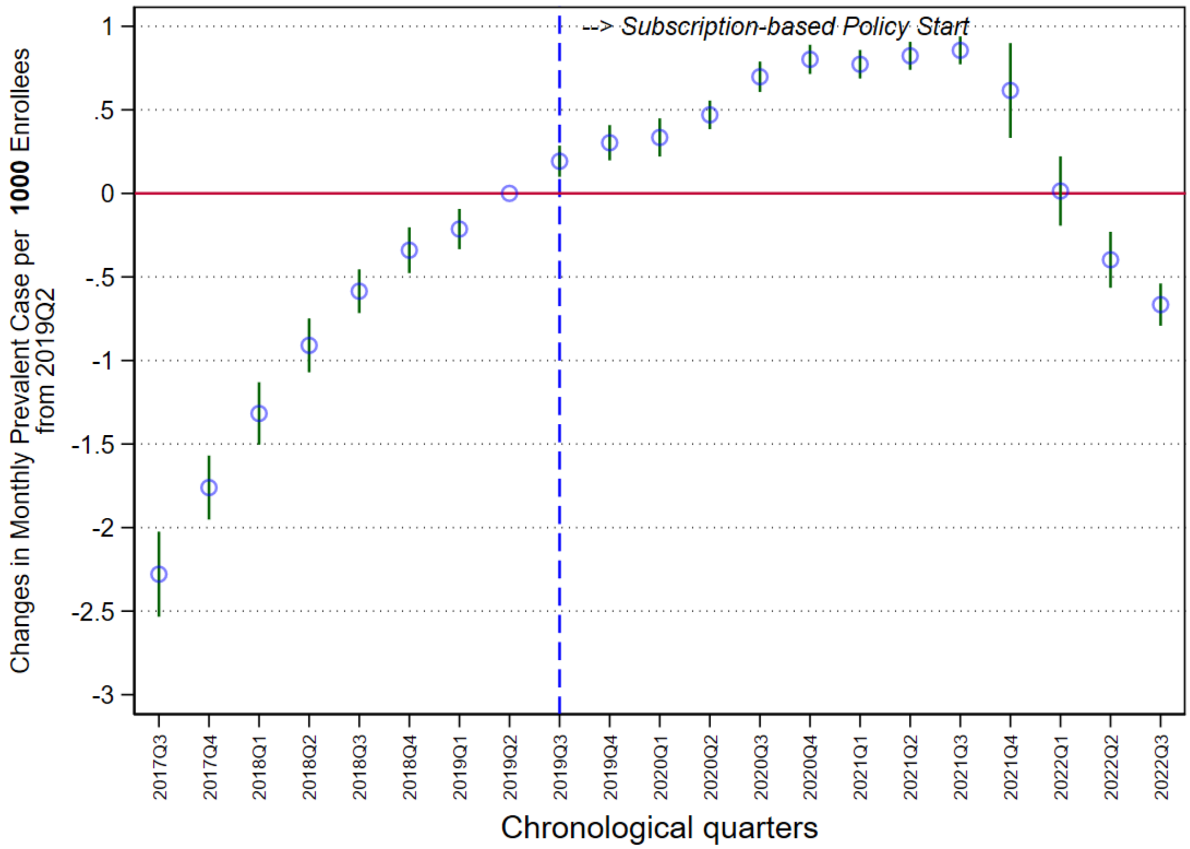

eFigure 2. Change in prevalent cases per 1000 enrollees compared to reference quarter (2019Q2)
Source - Authors' analysis of data from the Washington State All Payers Claims Database, 2025

© 2026 Tabah A et al. *JAMA Network Open*.

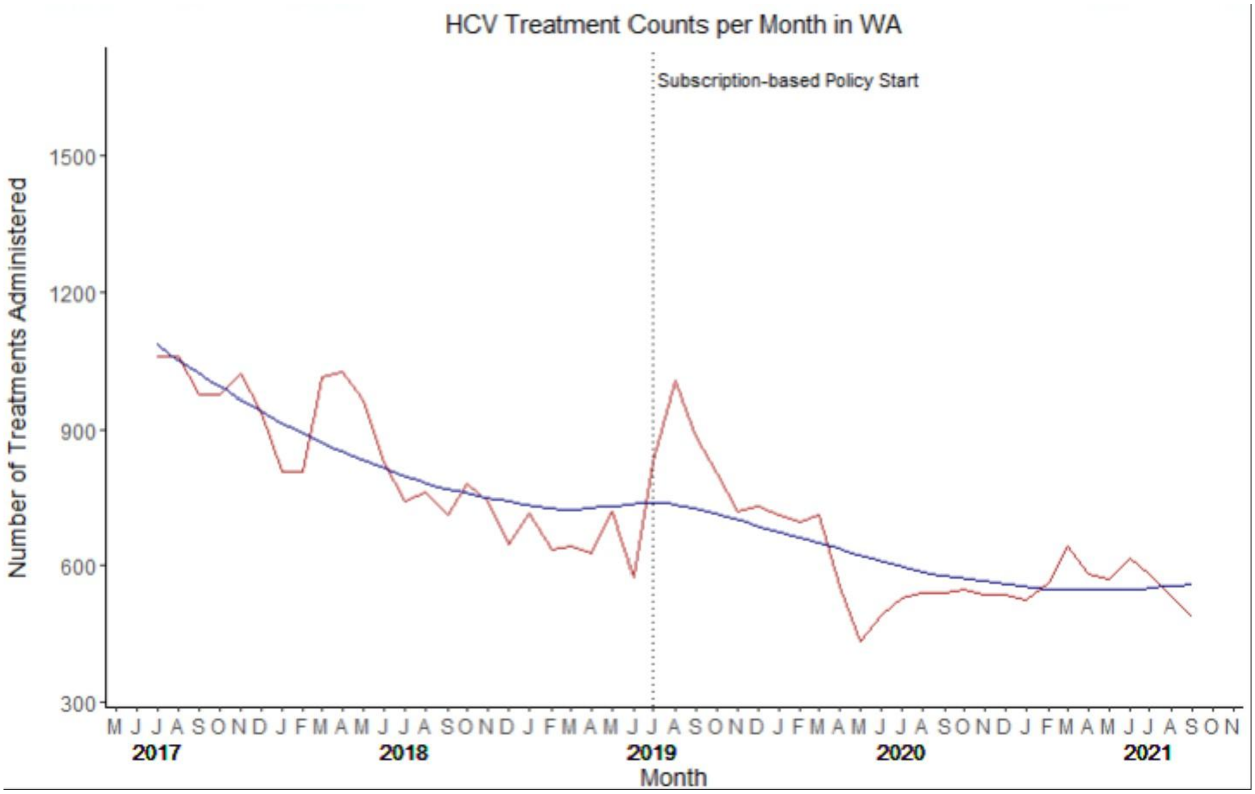

eFigure 3. Monthly counts of curative treatment administered in Washington state.
Source - Authors' analysis of data from the Washington State All Payers Claims Database, 2025

© 2026 Tabah A et al. *JAMA Network Open*.

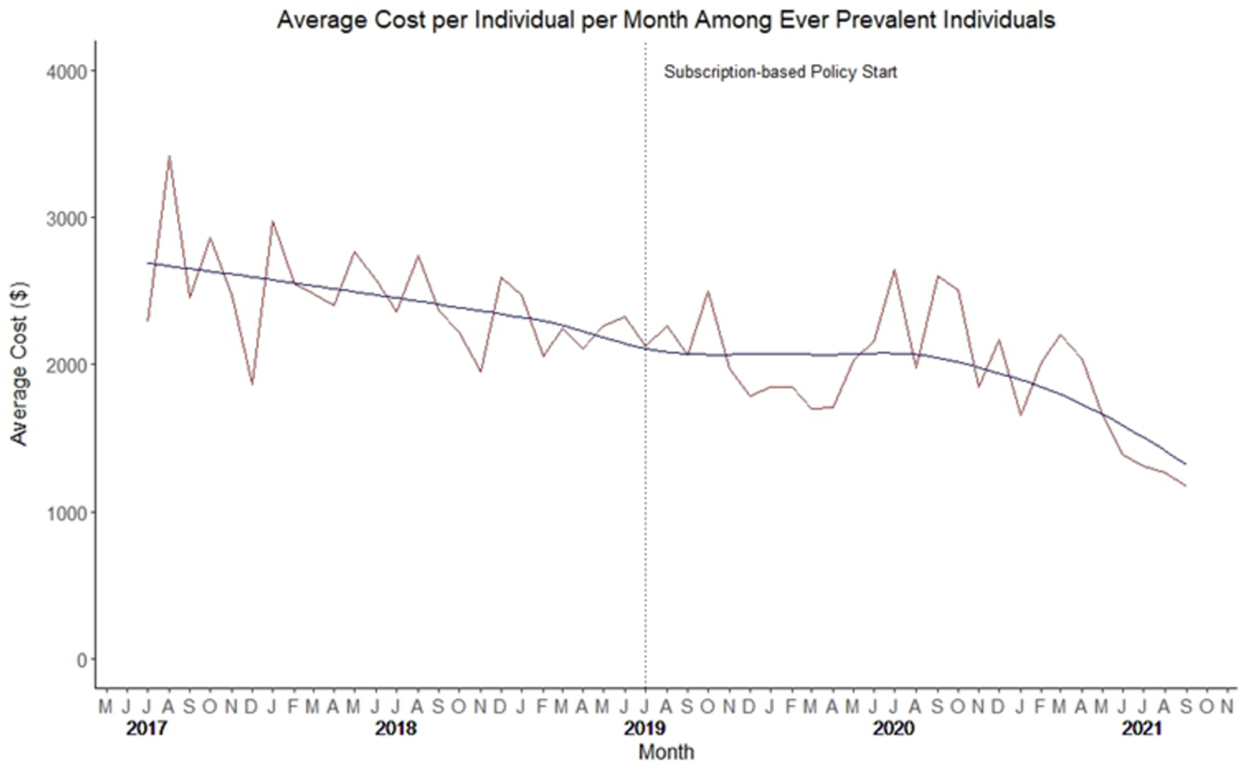

eFigure 4. Per-patient average total health care costs (without HCV direct-acting antiviral medication costs). Source - Authors' analysis of data from the Washington State All Payers Claims Database, 2025

© 2026 Tabah A et al. *JAMA Network Open*.
