# Supplementary material for: Trends in Utilization and Costs Following a Hepatitis C Elimination Initiative
Source: JAMA Netw Open. 2026 Feb 10;9(2):e2558714. doi: 10.1001/jamanetworkopen.2025.58714 (PMC12892153; doi:10.1001/jamanetworkopen.2025.58714)
Supplement: Supplement 2. — Data Sharing Statement [file jamanetwopen-e2558714-s002.pdf]

Data Sharing Statement

Tabah. Trends in Utilization and Costs Following a Hepatitis C Elimination Initiative. *JAMA Netw Open*. Published February 10, 2026. doi:10.1001/jamanetworkopen.2025.58714

Data
Data available: No

Additional Information
Explanation for why data not available: We cannot provide individual level data, but we can provide month level data/counts per our agreement with the APCD. Data dictionary is available.
